# Conventional technique versus no-touch technique in autogenous arteriovenous fistula: A meta-analysis

**DOI:** 10.1097/MD.0000000000044550

**Published:** 2025-09-19

**Authors:** Weigang Tang, Tong Li, Wei Jiang, Zhixia Wang, Xianping Li, Xiaoming Liu, Qing Luo, Macuo Wan, Wanxin Zhong, Zhuoma Caiji, Danzhi Xiangxiu, Wei Zhao, Fancheng Kong, Xiang Dan, Wei Xu

**Affiliations:** a Department of Nephrology, The Wujin Clinical College of Xuzhou Medical University, Changzhou, Jiangsu Province, China; b Jiangsu Key Laboratory of New Drug Research and Clinical Pharmacy, Xuzhou Medical University, Xuzhou, Jiangsu Province, China; c Department of Nephrology, Qinghai Red Cross Hospital, Xining, Qinghai Province, China; d Department of Nephrology, People’s Hospital of Hainan Tibetan Autonomous Prefecture, Hainan Tibetan Autonomous Prefecture, Qinghai Province, China.

**Keywords:** arteriovenous fistula, meta-analysis, no-touch

## Abstract

**Background:**

The comparative benefit of the no-touch technique versus the conventional technique for autogenous arteriovenous fistula (AVF) creation is still uncertain. Thus, our meta-analysis was conducted to compare the clinical outcomes between these 2 techniques.

**Methods:**

Databases, including PubMed, EMBASE, the Cochrane Library, the China National Knowledge Infrastructure, and the Wanfang database, were searched from inception to May 1, 2025. Eligible studies comparing conventional and no-touch techniques for AVF creation were included. The data were analyzed using Review Manager Version 5.3.

**Results:**

Six studies were included in the meta-analysis. There was no significant difference between the conventional technique group and the no-touch technique group with regards to the success rate of surgery (odds ratio [OR] 0.38, 95% confidence interval [CI] 0.09–1.60, *P* = .19), maturation rate at 3 months (OR 0.53, 95% CI 0.24–1.18, *P* = .12), or secondary patency rate within 6 months to 1 year (OR 0.41, 95% CI 0.17–0.99, *P* = .05). The primary patency rate from 6 months to 2 years in the no-touch technique group was greater than that in the conventional technique group (OR 0.46, 95% CI 0.31–0.68; *P* < .0001).

**Conclusion:**

Compared with conventional techniques, the no-touch technique may have a greater primary patency rate for AVF creation. However, the 2 techniques did not significantly differ in terms of the success rate of surgery, maturation rate, or secondary patency rate.

## 1. Introduction

Autogenous arteriovenous fistula (AVF) is recommended as the preferred vascular access for long-term hemodialysis.^[1–4]^ However, conventional AVF creation is associated with a high dysfunction rate and complications such as failure to mature, junta-anastomotic stenosis, and thrombosis.^[5–7]^ Previous studies have shown that conventional AFV surgery damages the vasa vasorum and causes ischemia and oxidative stress in the vessel wall, which in turn leads to the proliferation of vascular cells, stenosis of the venous wall, and AVF dysfunction.^[8,9]^

In recent years, Hӧrer introduced the no-touch technique for AVF creation.^[10]^ Some trials have compared conventional techniques with no-touch techniques in terms of factors such as AVF maturation and patency. At present, the comparative benefit of the no-touch technique versus the conventional technique in AVF creation remains uncertain. Thus, our meta-analysis was conducted to compare the clinical outcomes between the conventional technique and the no-touch technique in terms of AVF creation.

## 2. Materials and methods

### 2.1. Search strategy

We searched PubMed, Embase, the Cochrane Library, the China National Knowledge Infrastructure, and Wanfang from inception to May 1, 2025. The combined text and MeSH terms included no touching and arteriovenous fistula. In addition, the cited papers and relevant references were searched manually to identify eligible studies. There were no language restrictions. Ethical approval was not necessary because our meta-analysis is a statistical analysis based on previous literature.

### 2.2. Inclusion and exclusion criteria

The inclusion criteria were as follows: randomized controlled trials (RCTs) and observational studies; uremia patients who needed hemodialysis and established AVF with appropriate vascular conditions and no surgical contraindications^[1]^; studies that compared conventional techniques and no-touch techniques in AVF creation; and the endpoints of the review were the success rate of surgery, maturation rate, AFV primary patency rates, and AFV secondary patency rates.

The exclusion criteria were as follows: severe cardiac insufficiency or unsuitable vasculature for AVF creation; case series, comments, reviews; and lack of relevant outcome data.

### 2.3. Data extraction and quality assessment

Data were extracted independently by 2 investigators from China using standard data extraction forms. In the case of disagreement, a third investigator was consulted. We extracted data such as the first author, year of publication, location, study design, follow-up period, age, sex, sample size, incidence of hypertension and diabetes, vein size, artery size, size and method of anastomosis, successful rate of surgery (the successful rate of surgery was defined as the immediate success rate of surgery), maturation rate (AVF maturation was defined as venous diameter >0.6 cm and access flow >600 mL/min at 3 months after the operation^[1]^), and patency rate (primary patency was the interval from the time of access placement until any intervention designed to maintain or reestablish patency, access thrombosis, or the time of measurement of patency. Secondary patency was calculated from the time of vascular access creation until permanent access failure, regardless of the number of procedures required to maintain access patency for dialysis^[4]^). The Cochrane assessment tool was used to evaluate the quality of the RCTs.^[11]^ The Newcastle–Ottawa scale (NOS) was used to evaluate the quality of nonrandomized studies.^[12]^

### 2.4. Statistical analysis

We performed the data analysis by using Review Manager Version 5.3 (Cochrane Collaboration). Heterogeneity between studies was assessed by using *I*^2^ statistics. We considered *I*^2^ > 50% and *P* < .10 to imply significant heterogeneity. Homogeneous data were analyzed using the fixed-effects model. The heterogeneity of the data was analyzed using the random effects model. We presented categorical variables as odds ratios (ORs). Summary estimates and 95% confidence intervals (CIs) were calculated. A *P*-value <.05 was considered significant. Publication bias was analyzed using a sensitivity analysis.

## 3. Results

### 3.1. Study selection and characteristics

**Figure 1.**
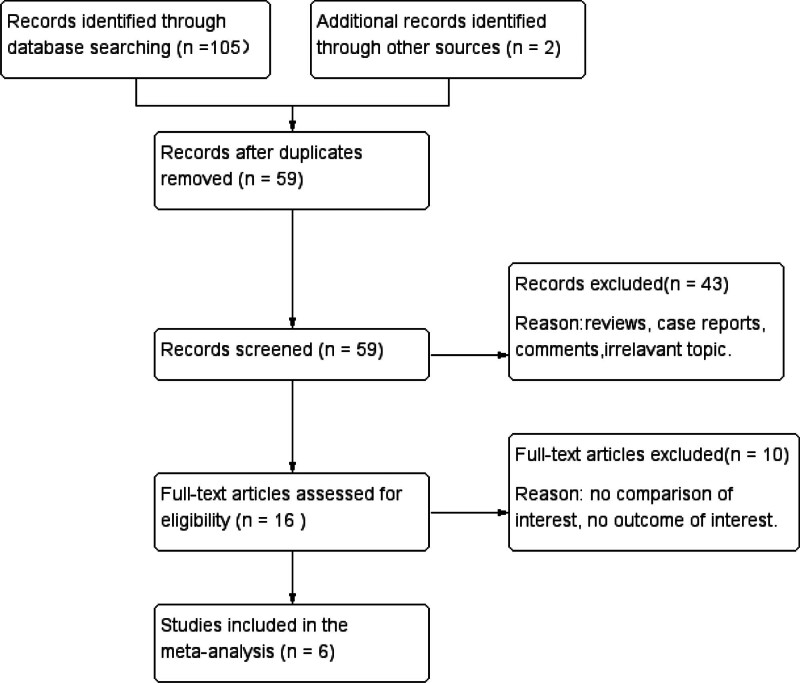
Flow diagram of the literature search.

A flow diagram of the selection process is shown in Figure 1. Six studies were included in the meta-analysis.^[13–18]^ A total of 360 patients were included in the conventional technique group, and 339 patients in the no-touch technique group. The follow-up period ranged from 6 months to 2 years. The baseline characteristics of these studies are listed in Table 1. The Cochrane assessment tool was used to evaluate the quality of the 2 RCTs. Two studies reported random sequence generation methods. One study reported allocation concealment with envelopes. Two studies did not report blinded allocation. Neither of the 2 studies had incomplete outcome data or selective reporting. The Cochrane assessment results are listed in Table 2. The NOS was used to evaluate the quality of 4 nonrandomized studies. All nonrandomized studies with scores of ≥6 points were considered high quality. The NOS assessment results are listed in Table 3.

**Table 1:** Characteristics of the included studies.

Study	Country	Design	Follow-up period	Sample size	Mean age (years)	Male/female	Diabetes (%)	Hypertension (%)	Vein size (mm)	Artery size (mm)	The size of anastomosis	The method of anastomosis
Sakr et al^[13]^	Egypt	RCT	6 months	Convention 40 No-touch 40	60.5 ± 9.2 56.1 ± 12.9	22/18 24/16	16 (40.0) 18 (45.0)	30 (75.0) 32 (80.0)	2.98 ± 0.31 2.97 ± 0.31	2.85 ± 0.27 2.76 ± 0.28	10–12 mm	End-to-side End-to-side
Hou et al^[15]^	China	Retrospective study	1 year	Convention 60 No-touch 40	54.2 ± 14.3 59.6 ± 18.0	39/21 26/14	17 (28.30) 16 (40.0)	13 (21.7) 3 (7.5)	2.98 ± 0.74 2.97 ± 0.74	2.25 ± 0.34 2.34 ± 0.42	-	End-to-side Functional end-to-side
Hastaoğlu et al^[14]^	Turkey	Observational study	1 year	Convention 95 No-touch 74	64.5 ± 11.3 63.1 ± 11.6	58/37 49/25	51 (53.7) 33 (44.6)	66 (69.5) 44 (61.1)	2.3 ± 0.2 2.2 ± 0.1	2.3 ± 0.2 2.3 ± 0.2	4–6 mm	End-to-side End-to-side
Ye et al^[16]^	China	RCT	1 year	Convention 89 No-touch 90	52.3 ± 14.9 56.1 ± 14.6	49/40 51/39	28 (31.5) 32 (35.5)	22 (24.7) 24 (26.6)	1.88 ± 0.57 1.81 ± 0.61	1.87 ± 0.54 1.85 ± 0.54	8 mm	End-to-side End-to-side
Wang et al^[17]^	China	Retrospective study	1 year	Convention 40 No-touch 63	60 ± 13 60 ± 17	23/17 39/24	20 (50.0) 23 (36.5)	–	2.59 ± 0.62 2.86 ± 0.72	2.57 ± 0.74 2.41 ± 0.61	5–10 mm	End-to-side Functional end-to-side
Jiao et al^[18]^	China	Retrospective study	2 year	Convention 36 No-touch 32	17–73 16–75	25/11 24/8	9 (25.0) 8 (25.0)	27 (75.0) 23 (71.9)	–	–	5–8 mm	End-to-side or end-to-end

RCT = randomized controlled trial.

**Table 2:** Quality assessment of randomized control trial.

Study	Random sequence generation	Allocation concealment	Blinding of participants and personnel	Incomplete outcome data	Selective reporting	Other bias
Sakr et al^[13]^	+	?	?	+	+	?
Ye et al^[16]^	+	+	?	+	+	?

The randomized control trial was evaluated using the Cochrane assessment tool: +, low risk of bias; ?, unclear risk of bias; −, high risk of bias.

**Table 3:** Quality assessment of nonrandomized studies.

Studies	Selection	Comparability	Outcome	Score
Hou et al^[15]^	★★★	★	★★★	7
Hastaoğlu et al^[14]^	★★★★	★	★★★	8
Wang et al^[17]^	★★	★	★★★	6
Jiao et al^[18]^	★★	★	★★★	6

The Cohort studies were evaluated using the Newcastle–Ottawa scale, which are comprised of the study of selection (representativeness of the exposed group, representativeness of the non exposed group, ascertainment of exposure, demonstration that outcome of interest was not present at start of study), group comparability (controls for the most important factor, controls for any additional factor), and outcome measures (assessment of outcome, was follow-up long enough for outcomes to occur, adequacy of follow-up of cohorts); a total of 9 points. ★, 1 point.

### 3.2. Meta-analysis results

#### 3.2.1. Surgery rate

**Figure 2.**
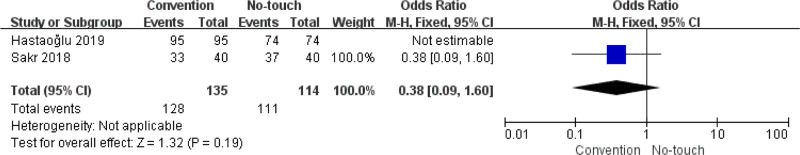
Forest plots comparing succesful rate of surgery between conventional technique and no-touch technique. CI = confidence interval.

Data reporting a successful rate of surgery were reported in 2 articles: a ratio of 128/135 (94.8%) for the conventional technique group and 111/114 (97.3%) for the no-touch technique group. There was no significant difference between the conventional technique group and the no-touch technique group with regards to the success rate of surgery (OR 0.38, 95% CI 0.09–1.60; *P* = .19) (Fig. 2).

#### 3.2.2. Maturation rate

**Figure 3.**
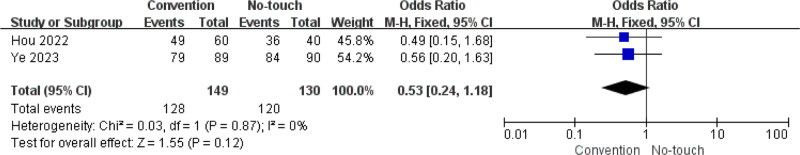
Forest plots comparing maturation rate at 3 months between conventional technique and no-touch technique. CI = confidence interval.

Data regarding the maturation rate at 3 months were reported in 2 articles: 128/149 (85.9%) for the conventional technique group and 120/130 (92.3%) for the no-touch technique group. There was no significant difference in the maturation rate at 3 months between the conventional technique group and the no-touch technique group (OR 0.53, 95% CI 0.24–1.18; *P* = .12) (Fig. 3).

#### 3.2.3. Primary patency rate

**Figure 4.**
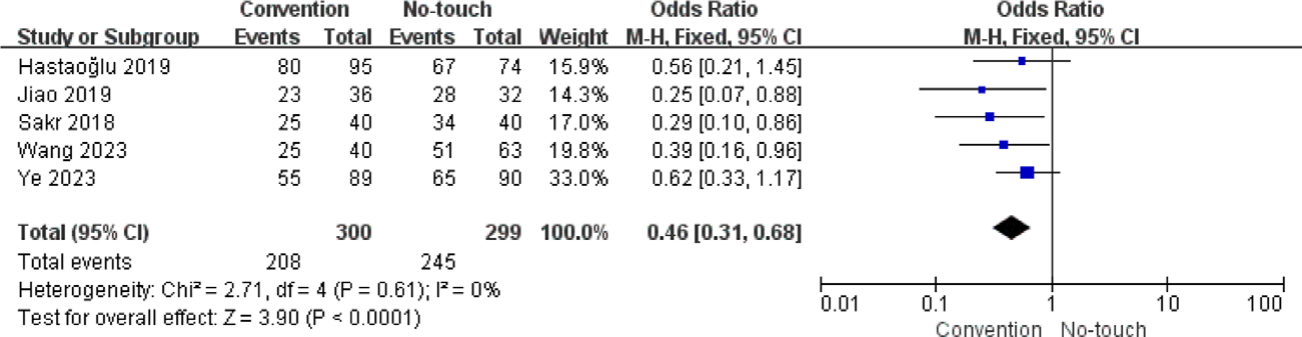
Forest plots comparing primary patency within 6 months to 2 year between conventional technique and no-touch technique. CI = confidence interval.

Data showing the primary patency rate within 6 months to 2 years were reported in 4 articles: 208/300 (69.3%) in the conventional technique group and 245/299 (81.9%) in the no-touch technique group. Compared with the conventional technique, the no-touch technique resulted in significantly better primary patency rates (OR 0.46, 95% CI 0.31–0.68, *P* < .0001) (Fig. 4), with the upper confidence limit (0.68) remaining below the null threshold (OR 1.0), demonstrating its clear superiority.

#### 3.2.4. Secondary patency rate

**Figure 5.**
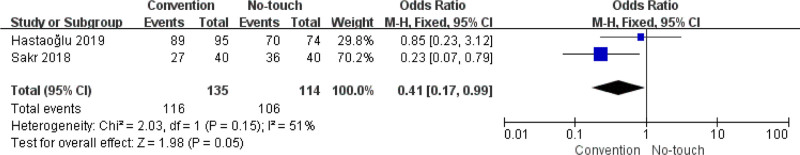
Forest plots comparing secondary patency within 6 months to 1 year between conventional technique and no-touch technique. CI = confidence interval.

Data showing the secondary patency rate within 6 months to 1 year were reported in 2 articles: 116/135 (85.9%) in the conventional technique group and 106/114 (93.0%) in the no-touch technique group. There was no significant difference between the conventional technique group and the no-touch technique group regarding the secondary patency rate from 6 months to 1 year (OR 0.41, 95% CI 0.17–0.99; *P* = .05) (Fig. 5).

### 3.3. Sensitivity analyses

Sensitivity analyses were used to judge the dependability of the meta-analysis results of the outcomes. For the outcome of the secondary patency rate within 6 months to 1 year, we excluded the study of Hastaoğlu 2019; the meta-analysis results revealed that the secondary patency rate within 6 months to 1 year was higher in the no-touch technique group than in the conventional technique group. For other outcomes, the meta-analysis results showed no change when we deleted 1 study at a time.

## 4. Discussion

A well-functioning AVF is the key to performing efficient hemodialysis. The risk factors affecting AVF function include sex, age, diabetes mellitus, cardiovascular disease, and vascular diameter,^[19–21]^ which are difficult to change. However, we can improve surgical methods to enable the AVF to remain functional for a longer time. Our meta-analysis revealed that the primary patency rate in the no-touch technique group was greater than that in the conventional technique group, but there was no significant difference between the conventional technique group and the no-touch technique group with respect to the success rate of surgery, maturation rate, or secondary patency rate.

Compared with the conventional technique, the no-touch technique has greater advantages in terms of the primary AVF creation rate. In coronary artery bypass grafting, some studies have reported that the no-touch harvesting technique has superior outcomes, including improved long-term patency rates and slower the progression of atherosclerosis.^[22]^ The possible reasons are as follows.^[13–18,22,23]^ First, the no-touch technique not only preserves the outer membrane and peripheral fat structure of the vein but also avoids spasm of the vein and damage to its walls. This enables the cellular structure and functions of the entire vascular wall to be maintained relatively completely, and it also better preserves the vasa vasorum, which can reduce fibrous proliferation and stenosis of the vein and improve the long-term patency rate.^[17,23–25]^ Second, the retained perivenous fat tissue can exert a buffering effect on the elevated blood pressure in the AVF, which reduces the degree of pressure injury to the vein.^[22,25,26]^ Third, the retained perivenous fat tissue can generate vasodilator factors.^[25,27,28]^ Fourth, the no-touch technique had a good prognosis related to improved fistula hemodynamics.^[29]^ In addition, the no-touch technique had a slightly higher secondary patency rate than did the traditional technique, but the difference was not statistically significant. Notably, the assessment of the secondary patency rate may be interfered with by intervention measures such as balloon dilation, which may not reflect the original effect of the surgical technique.^[1]^

There were several limitations in our meta-analysis. First, there were some differences in the operation methods used with no-touch technology among the included studies. In the studies of Wang and Hou, no-touch technology adopted functional end-to-side anastomosis, which might cause publication bias. For the results of the primary patency rate, we used sensitivity analysis to judge the dependability of these meta-analysis results because the research data of Wang and Hou were included. After the studies of Wang or Hou were excluded, no changes were found in the meta-analysis results for the primary patency rate. Second, the time points of the primary and secondary patencies in the included studies were different, which might cause publication bias. Third, other factors, such as age, diabetes status, and high blood pressure, might also lead to publication bias. Fourth, the number of studies included in our meta-analysis was insufficient. To further confirm these results, larger multicentre RCTs comparing these 2 surgical methods are necessary.

## 5. Conclusions

Compared with conventional techniques, the no-touch technique may have a higher primary patency rate for AVF creation. However, the 2 techniques did not significantly differ in terms of the success rate of surgery, maturation rate, or secondary patency rate. To further confirm this conclusion, larger multicenter RCTs comparing the 2 AVF surgical methods are necessary.

## Author contributions

**Conceptualization:** Weigang Tang, Tong Li, Wei Xu.

**Data curation:** Weigang Tang, Tong Li.

**Investigation:** Wei Jiang, Zhixia Wang, Xianping Li, Wei Xu.

**Methodology:** Weigang Tang, Wei Xu.

**Visualization:** Wei Zhao, Fancheng Kong, Xiang Dan.

**Writing – original draft:** Xiaoming Liu, Qing Luo, Macuo Wan, Wanxin Zhong, Zhuoma Caiji, Danzhi Xiangxiu, Wei Xu.

**Writing – review & editing:** Wei Xu.

Supplemental digital content “Supplementary file.Search strategy” is available for this article (https://links.lww.com/MD/Q26).

## Supplementary Material


