# Supplementary material for: Conventional technique versus no-touch technique in autogenous arteriovenous fistula: A meta-analysis
Source: Medicine (Baltimore). 2025 Sep 19;104(38):e44550. doi: 10.1097/MD.0000000000044550 (PMC12459549; doi:10.1097/MD.0000000000044550)
Supplement: Supplementary file 1 [file medi-104-e44550-s001.docx]

**Search strategy of PubMed**

1#: (nitrogen-containing bisphosphonate [MeSH Terms]) OR (nitrogen-containing bisphosphonate [Title/Abstract])

2#: (minodronate [MeSH Terms]) OR (minodronate [Title/Abstract]) OR (minodronic acid [Title/ Abstract])

3#: (alendronate[MeSH Terms]) OR (alendronate[Title/Abstract]) OR (alendronic acid [Title/ Abstract])

4#: (risedronate[MeSH Terms]) OR (risedronate[Title/Abstract]) OR(risedronic acid [Title/ Abstract])

5#: (ibandronate[MeSH Terms]) OR (ibandronate[Title/Abstract]) OR(ibandronic acid [Title/ Abstract])

6#: (zoledronate[MeSH Terms]) OR (zoledronate[Title/Abstract]) OR (zoledronic acid [Title/ Abstract])

7#: (pamidronate[MeSH Terms]) OR (pamidronate[Title/Abstract]) OR (pamidronic acid [Title/ Abstract])

8#: (vascular calcification[MeSH Terms]) OR (vascular calcification[Title/Abstract]) OR (Vascular Calcinosis[Title/Abstract])OR (Vascular Calcinoses[Title/Abstract])

9#: 1# OR 2# OR 3# OR 4# OR 5# OR 6# OR 7#

10#: 8# AND 9#

**Search strategy of Embase**

1#: 'nitrogen-containing bisphosphonate':ti,ab,kw OR 'nitrogen-containing bisphosphonate'/exp OR 'minodronic acid':ti,ab,kw OR 'minodronate':ti,ab,kw OR 'minodronate'/exp OR 'alendronic acid':ti,ab,kw OR 'alendronate':ti,ab,kw OR 'alendronate'/exp OR 'risedronic acid':ti,ab,kw OR 'risedronate':ti,ab,kw OR 'risedronate'/exp OR 'ibandronic acid':ti,ab,kw OR 'ibandronate':ti,ab,kw OR 'ibandronate'/exp OR 'zoledronic acid':ti,ab,kw OR 'zoledronate':ti,ab,kw OR 'zoledronate'/exp OR 'pamidronic acid':ti,ab,kw OR 'pamidronate':ti,ab,kw OR 'pamidronate'/exp

2#: 'Vascular Calcinoses':ti,ab,kw OR 'Vascular Calcinosis':ti,ab,kw OR 'Vascular Calcinoses':ti,ab,kw OR 'Vascular Calcinoses'/exp

3#: 1# AND 2#

**Search strategy of Embase**

1#: (nitrogen-containing bisphosphonate):ti,ab,kw OR (minodronic acid):ti,ab,kw OR (minodronate):ti,ab,kw OR (alendronic acid):ti,ab,kw OR (alendronate):ti,ab,kw OR (risedronic acid):ti,ab,kw OR (risedronate):ti,ab,kw OR (ibandronic acid):ti,ab,kw OR(ibandronate):ti,ab,kw OR (zoledronic acid':ti,ab,kw OR (zoledronate):ti,ab,kw OR (pamidronic acid):ti,ab,kw OR (pamidronate):ti,ab,kw

2#:MeSH descriptor: [nitrogen-containing bisphosphonate] explode all trees

3#:MeSH descriptor: [minodronate] explode all trees

4#:MeSH descriptor: [risedronate] explode all trees

5#:MeSH descriptor: [ibandronate] explode all trees

6#:MeSH descriptor: [zoledronate] explode all trees

7#:MeSH descriptor: [pamidronate] explode all trees

8#: MeSH descriptor: [vascular calcification] explode all trees

9#: (vascular calcification):ti,ab,kw OR (Vascular Calcinosis):ti,ab,kw OR (Vascular Calcinoses): ti,ab,kw

10#: 1# OR 2# OR 3# OR 4# OR 5# OR 6# OR 7#

11#: 8# OR 9#

12#:10# OR 11#

**Search strategy of CNKI**

1#: （主题：含氮双膦酸盐）OR（全文：含氮双膦酸盐(精确)）OR（主题：米诺膦酸）OR（全文：米诺膦酸(精确)）OR（主题：阿仑膦酸）OR（全文：阿仑膦酸(精确)）OR（主题：利塞膦酸）OR（全文：利塞膦酸(精确)）OR（主题：伊班膦酸）OR（全文：伊班膦酸(精确)）OR（主题：唑来膦酸）OR（全文：唑来膦酸(精确)）OR（主题：帕米膦酸）OR（全文：帕米膦酸(精确)）

2#: （主题：血管钙化）OR（全文：血管钙化(精确)）OR（全文：血管钙质沉着(精确)）

3#: 1# AND 2#

**Search strategy of Wanfang database**

1#: 主题:(含氮双膦酸盐) or 全部:(含氮双膦酸盐) or 主题:(米诺膦酸) or 全部:(米诺膦酸) or 主题:(阿仑膦酸) or 全部:(阿仑膦酸) or主题:(伊班膦酸) or 全部:(伊班膦酸) or 主题:(唑来膦酸) or 全部:(唑来膦酸) or 主题:(帕米膦酸) or 全部:(帕米膦酸)

2#: 主题:(血管钙化) or 全部:(血管钙化)

3#: 1# AND 2#
